# Microsimulation of an educational attainment register to study record linkage quality.

**DOI:** 10.23889/ijpds.v7i3.1848

**Published:** 2022-08-25

**Authors:** Rainer Schnell, Severin Weiand

**Affiliations:** 1 University of Duisburg-Essen

Population covering educational attainment registers have been proven helpful for planning and research concerning educational efforts. Regular linking of different databases is needed to build and update such a register. Without unique national identification numbers, record linkage must be based on quasi-identifiers such as names, date of birth and sex. High-quality record linkage require the unique identification of persons. Therefore, available identifiers should be sufficient for unique identification despite missing identifiers for some cases. Redundant identifiers can achieve this goal. However, the data protection principle of data minimization, as recommended in the European General Data Protection Regulation, aims to avoid additional data if possible for the given purpose. Therefore, a ministry commissioned a simulation study to inform legislators on the minimum set of identifiers needed for a national register. A microsimulation of the population consisting of nearly 20 million people was implemented to generate data on accumulating changes and errors in identifiers over ten simulated years. The simulation covered, for example, international migration, regional mobility, marriages, school careers and mortality. Each event triggered changes of identifiers according to specified error probability models. The resulting data were linked by different record-linkage procedures. Linkage quality and linkage bias dependent on the available identifiers were assessed. We report on the design of the simulation study, the linkage results and recommendations for the minimum set of identifiers. The results may be helpful for the design of other population covering registers.

